# An in vivo assessment of adriamycin-loaded albumin microspheres.

**DOI:** 10.1038/bjc.1992.79

**Published:** 1992-03

**Authors:** J. A. Goldberg, N. Willmott, D. J. Kerr, C. Sutherland, C. S. McArdle

**Affiliations:** University Department of Surgery, Royal Infirmary, Glasgow, UK.

## Abstract

**Images:**


Br. J. Cancer (1992), 65, 393-395 © Macmillan Press Ltd., 1992

### An *in vivo* assessment of Adriamycin-loaded albumin microspheres

J.A. Goldberg[1], N. Willmott[2], D.J. Kerr[3], C. Sutherland[4] & C.S. McArdle[1]

*University Departments of [1]Surgery and [4]Pathology, Royal Infirmary, Glasgow, [2]Department of Pharmacy, University of Strathclyde, Glasgow, [3]Cancer Research Campaign Department of Clinical Oncology, University of Glasgow, UK.*

The results of systemic chemotherapy for treatment of many of the solid tumours are poor. Regional chemotherapy is an attractive concept which aims to improve tumour drug exposure and reduce systemic toxicity by delivering the cytotoxic in concentrated from directly to the tumour-bearing organ, via its arterial supply.

In the absence of new agents with specific activity and acceptable toxicity, attention has turned to methods of enhancing the effect of drugs which are currently available. Novel formulations of established cytotoxics can be synthesised which are particularly applicable to regional delivery. Willmott and co-workers (1985) have described Adriamycin loaded albumin microspheres – particles with a diameter of 20–40 μm which are made by glutaraldehyde stabilisation of an oil in water emulsion.

Early animal experiments with regionally administered albumin microspheres confirmed almost complete embolisation of particles within a target organ, and showed that the microspherical formulation was associated with little systemic drug exposure (McArdle *et al.*, 1988).

The aim of the present study was to assess the anti-tumour effect of Adriamycin-loaded albumin microspheres given regionally in a rat tumour model.

Aggregate-free suspensions of Adriamycin-loaded microspheres were made by a technique based on that of Lee and colleagues, 1981. The particles were filtered to obtain a diameter of between 20 and 40 μm, 50% weight average (Willmott *et al.*, 1985).

The quantity of Adriamycin within the particles was evaluated by HPLC (Cummings & Willmott, 1985). Two milligrams of microspheres contained approximately 10 μg of 'native' Adriamycin, and 20 μg of covalently bound drug.

Three day old hepatic tumour implants of Walker 256 (70 mg) in Sprague-Dawley rats (600–700 g) were used in this study (Ackerman *et al.*, 1969). Model characterisation studies using hepatic arterial and portal venous injections of radiolabelled microspheres had shown that the tumour vasculature was derived almost exclusively from the hepatic artery and that the arterial perfusion of tumours was proportionally higher than that of the surrounding normal liver parenchyma. Arterio-venous shunting was minimal (Goldberg, 1990).

Four groups of six tumour-bearing animals were starved for 12 h; under ether anaesthesia, all underwent laparotomy for temporary cannulation of the gastroduodenal artery, which allowed infusion of one of the following via the hepatic arterial stream:

I Intra-arterial normal saline (50 μl).

II Intra-arterial empty albumin microspheres (2 mg in 50 μl).

III Intra-arterial Adriamycin 30 μg in 50 μl.

IV Intra-arterial Adriamycin loaded microspheres (2 mg containing approximately 30 μg of Adriamycin in 50 μl).

The animals were recovered and allowed free access to food and water.

On the seventh day after tumour implantation, the animals were culled, the tumours excised from the liver parenchyma and weighed blind. Both tumour and normal liver were preserved for histological and fluorescent microscopic detection of Adriamycin.

The tumour weights of each group at cull were compared using the Mann-Whitney test.

The results are summarised in Table I. The mean tumour weight at cull was significantly lower in the group of animals treated with Adriamycin loaded albumin microspheres.

Pathological assessment showed no obvious differences between the groups in terms of the relative amount of remaining viable tissues within the tumour nidus. Fragments of microspheres could be seen within tumour from the animals in groups II and IV (Figure 1).

Fluorescent microscopy detected considerable amounts of Adriamycin in the liver and tumour of animals treated with drug-loaded microspheres (Figure 2), whereas no drug remained in the tissues of animals treated with drug in solution. Intracellular Adriamycin was demonstrated in the tumour and liver of animals which had received the drug loaded particles.

The systemic treatment of many solid tumours by chemotherapy has yielded disappointing results. This has led to increasing interest in regional chemotherapy, but the theoretical advantages of arterial drug administration are not always apparent in practice.

The magnitude of the gain in regional selectivity depends upon factors such as saturation of the mechanism for drug extraction by the target organ, and the presence of arteriovenous shunts. The advantage of a regional approach to drug aministration is difficult to assess directly in patients: pharmacokinetic studies create a broad overview of systemic exposure, but yield no information about tumour drug levels or metabolism.

The data presented here suggest that the drug loaded particles are more potent in their anti-tumour effect than the drug in solution. The demonstration of intracellular drug in target tissue 4 days after microsphere infusion suggests that Adriamycin-loaded albumin microspheres have 'slow-release' properties *in vivo* which sustain high target-tissue drug levels for much longer than those achieved with regional administration of the drug in solution.

There is other *in vivo* evidence to suggest that native Adriamycin incorporated within microspheres might indeed

**Table I** Mean weight of implant 4 days following treatment

| *Group* | *n* | *Mean tumour weight (grams)* | *SD* |
|---|---|---|---|
| I | 6 | 0.73 | 0.16 |
| II | 6 | 1.01 | 0.27 |
| III | 6 | 0.74 | 0.12 |
| IV | 6 | 0.45 | 0.08[a] |

Group I: IA Normal saline; Group II: IA Albumin microspheres; Group III: IA Adriamycin in solution; Group IV: IA Adriamycin-loaded albumin microspheres. [a]$P < 0.05$, Mann-Whitney.

Correspondence: J.A. Goldberg, Department of Surgery, Victoria Infirmary, Glasgow, UK.
Received 4 March 1991; and in revised form 3 October 1991.

**Figure 1** Histological section (haemotoxylin and eosin) of Walker 256 intrahepatic implant. This animal had been treated with an injection of Adriamycin-loaded albumin microspheres 4 days prior to cull. Intra-arterial fragments of drug-loaded particles can be seen within viable tumour in the centre of the field. Tumour tissue is seen on the left (necrotic) and centre (viable) of the field; normal liver parenchyma is seen on the right of the field.

**Figure 2** Fluorescent micrograph of tumour tissue from an animal treated with Adriamycin-loaded albumin microspheres 4 days prior to cull. Intravascular fragments of drug-loaded particles can be seen containing a high concentration of Adriamycin, resulting in the intense fluorescence. However, the presence of an overall background fluorescence throughout the section shows that drug is present within the tissue at an intracellular level. In fluorescent micrographs of tumour from animals treated with a similar dose of drug in solution, no drug could be detected.

be more potent than the free drug (Willmott & Cummings, 1987). It has been shown that native Adriamycin in microspherical form is metabolised in tumour tissue via a reductive pathway, raising the possibility of reactive drug intermediates. More recent work, however, suggests that for Adriamycin, it is the sustained release properties of microspheres which are important for anti-tumour activity, rather than induction of reductive drug metabolism (Willmott *et al.*, 1990). Sustained release and increased exposure to Adriamycin is clearly illustrated in Figures 1 and 2.

These reports support other work which suggests that drug delivery systems can confer new and unexpected modes of action of drugs incorporated within particles, as well as changing drug disposition (Tritton & Yee, 1982; Rogers & Tokes, 1984; Mehta *et al.*, 1984). It is this aspect of microsphere bound regional chemotherapy which has the greatest potential because it is possible that the potency of existing cytotoxic drugs may be increased.

The myocardial toxicity associated with systemic administration of Adriamycin limits the cumulative dose in patients to 450 mg $m^{-2}$. However, a microsphere bound formulation of Adriamycin administered regionally, which greatly reduced systemic exposure and achieved improved response rates at lower drug dosage, would increase its therapeutic potential and allow dose escalation.

This study has shown that Adriamycin-loaded albumin microspheres can suppress tumour growth in an animal model. The use of regionally administered particulate drug formulations may offer a new approach in the management of solid tumours refractory to treatment by conventional therapy.

We are grateful to Mr Douglas Bell, Mr David McMurdo, and their technical staff for advice and practical assistance with this work.

We are also indebted to Mr Alan Law (Office International, UK) for his loan of computer equipment.

This work was supported by Cancer Research Campaign and Medical Research Council, United Kingdom, and Association for International Cancer Research.

**References**

ACKERMAN, N.B., LEIN, W.M., KONDI, E.S. & SILVERMAN, N.A. (1969). The blood-supply of experimental liver metastases I. The distribution of hepatic artery and portal vein blood to small and large tumours. *Surgery*, **66**, 1067.

GOLDBERG, J.A. (1990). *Aspects of hepatic arterial chemotherapy in the treatment of colorectal liver metastases*. Thesis: University of Birmingham, UK.

MCARDLE, C.S., LEWI, H., HANSELL, D., KERR, D.J., MCKILLOP, J. & WILLMOTT, N. (1988). Cytotoxic-loaded albumin microspheres: a novel approach to regional chemotherapy. *Br. J. Surg.*, **75**, 132.

MEHTA, R., LOPEZ-BERESTEIN, G., HOPFER, R., MILLS, K. & JULIANO, R.L. (1984). Liposomal amphotericin-B is toxic to fungal cells but not to mammalian cells. *Biochem. Biophys. Acta*, **770**, 230.

ROGERS, V.E. & TOKES, Z.A. (1984). Novel mode of cytotoxicity obtained by coupling inactive anthracycline to a polymer. *Biochem. Pharmacol.*, **33**, 605.

TRITTON, T.R. & YEE, G. (1982). The anticancer agent Adriamycin can be actively cytotoxic without entering cells. *Science*, **217**, 248.

WILLMOTT, N., CUMMINGS, J., STUART, J.F.B. & FLORENCE, A.T. (1985). Adriamycin-loaded albumin microspheres: preparation in vivo distribution and release in the rat. *Biopharm. & Drug Dis-posit.*, **6**, 91.

WILLMOTT, N. & CUMMINGS, J. (1987). Increased anti-tumour effect of Adriamycin-loaded microspheres is associated with anaerobic bioreduction of drug in tumour tissue. *Biochem. Pharmacol.*, **36**, 521.

WILLMOTT, N., CUMMINGS, J., MARLEY, E. & SMYTH, J.F. (1990). Relationship between reductive drug metabolism in tumour tissue of anthracyclines in microspherical form and anti-tumour activity. *Biochem. Pharmacol.*, **39**, 1055.


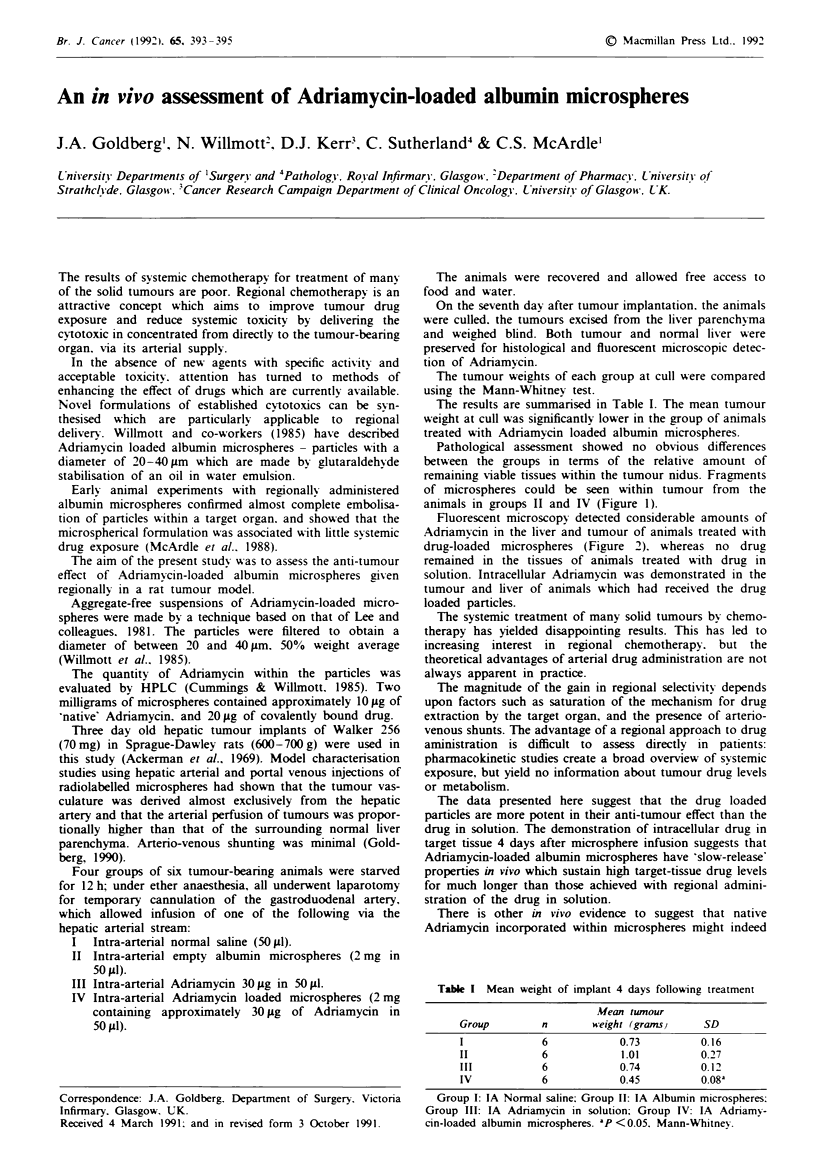

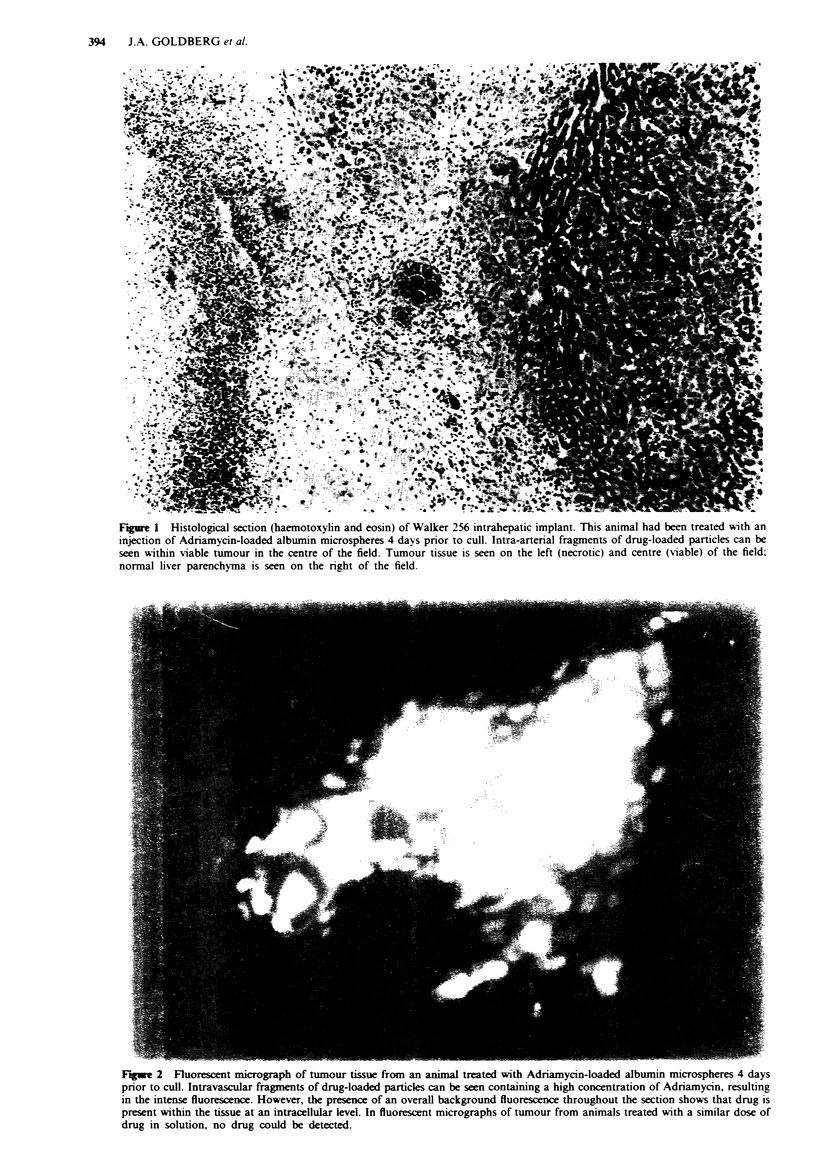

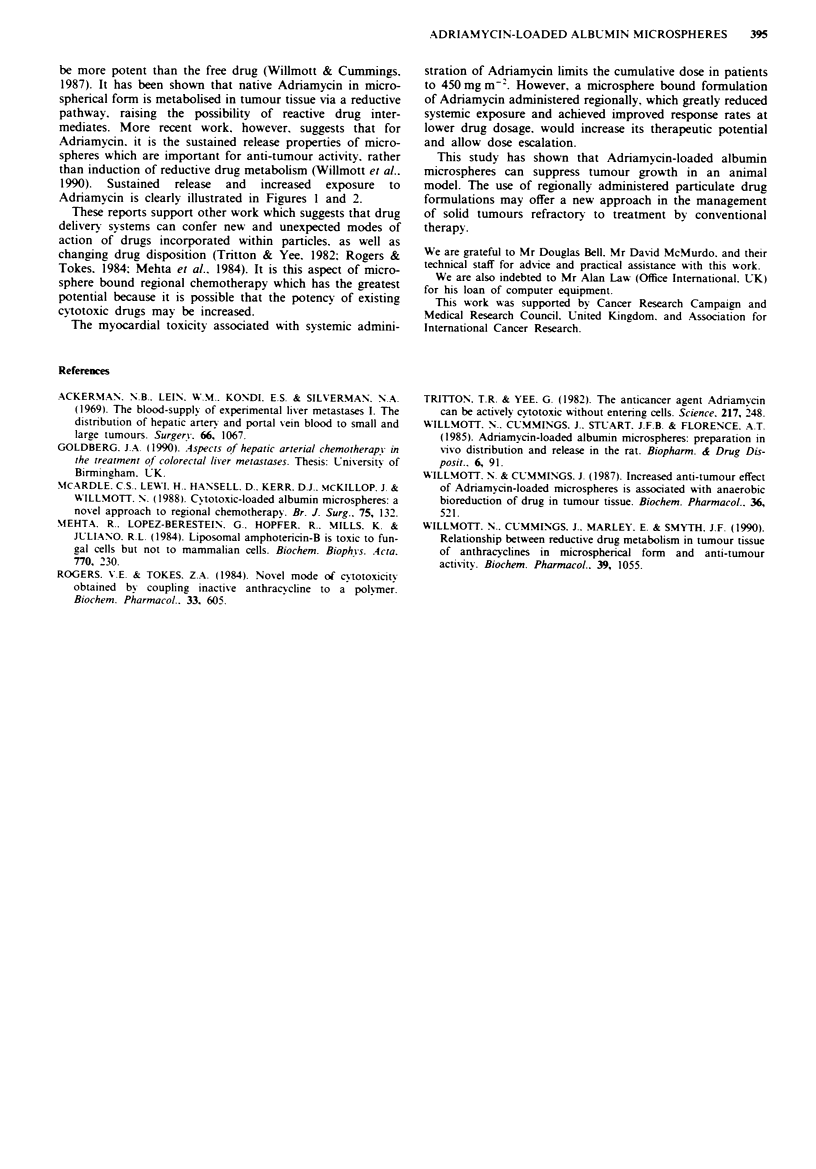


## References

[OCR_00228] Ackerman N. B., Lien W. M., Kondi E. S., Silverman N. A. (1969). The blood supply of experimental liver metastases. I. The distribution of hepatic artery and portal vein blood to "small" and "large" tumors.. Surgery.

[OCR_00240] McArdle C. S., Lewi H., Hansell D., Kerr D. J., McKillop J., Willmott N. (1988). Cytotoxic-loaded albumin microspheres: a novel approach to regional chemotherapy.. Br J Surg.

[OCR_00241] Mehta R., Lopez-Berestein G., Hopfer R., Mills K., Juliano R. L. (1984). Liposomal amphotericin B is toxic to fungal cells but not to mammalian cells.. Biochim Biophys Acta.

[OCR_00247] Rogers K. E., Tökés Z. A. (1984). Novel mode of cytotoxicity obtained by coupling inactive anthracycline to a polymer.. Biochem Pharmacol.

[OCR_00254] Triton T. R., Yee G. (1982). The anticancer agent adriamycin can be actively cytotoxic without entering cells.. Science.

[OCR_00261] Willmott N., Cummings J. (1987). Increased anti-tumor effect of adriamycin-loaded albumin microspheres is associated with anaerobic bioreduction of drug in tumor tissue.. Biochem Pharmacol.

[OCR_00267] Willmott N., Cummings J., Marley E., Smyth J. F. (1990). Relationship between reductive drug metabolism in tumour tissue of anthracyclines in microspherical form and anti-tumour activity.. Biochem Pharmacol.

